# The Garfield Doctors’ Bills

**Published:** 1882-07-20

**Authors:** 


### *THE GARFIELD DOCTORS' BILLS.*

We copy from a newspaper the following extract from Senator Vest's address, relative to the fees in the Garfield case:

"If anything is established by human testimony, if anything is creditable in regard to this case, it is that the late lamented President of the United States was—I will not use the word 'butchered'—but unquestionably malpractice was had upon him in his last illness, producing his death. It is admitted by the medical authorities who had charge of the case, and the autopsy put it beyond any sort of question, that he was treated for a pus cavity, while the wound which hurried him to the grave never for a moment came under the attention of those eminent surgeons. I allude to this in no spirit of malevolence; but I allude to it as a matter of legitimate discussion, to show that these gentlemen, from a medical and scientific point of view, are not entitled to the enormous fees which they now bring as a claim against the Government of the United States."

We do not propose to enter into an argument to defend the course and practice of the medical gentlemen who treated President Garfield; enough has already been said on the subject. As a journalist we wish to place a protest upon record against the severe and unjustifiable abuse of the medical men in this case, based upon the fact that they failed to trace the course of the ball. We insist that all the surgeons in the world, had they been there, could not have traced the devious track of the ball by any means now known to surgery; and if they could have done so, and located it in the exact spot where the autopsy found it, nothing could have been done to remove it or to change the result. That the case was a fatal one from the beginning there is now no doubt, and as little doubt that his life was prolonged and his sufferings greatly alleviated by the physicians. For this, and for the care and dilligence they bestowed upon the illustrious patient, they are entitled to the gratitude of the American people.

As to the fees, we do not favor any extortionate charges; nor do we understand that the medical men have made any charge—the matter being referred to a committee of Congress.

If so liberal a fee were to be conferred upon some Government agent, or some shrewd and distinguished attorney acting for the Government, in some matter of public interest, it would be thought legitimate and right; but when a member of the medical profession happens once in a century to fall in the way of receiving a bounty, then it is pronounced an outrage and a shame. Gen. Grant, already possessed of a large fortune, and loaded down with honors, has had bounties lavished upon him; and Mrs. Garfield was made the recipient of hundreds of thousands, and no word of conplaint is heard; but now that two or three conscien-cious and faithful medical men, bearing the unjust censure and abuse of half the nation, are to receive a somewhat liberal fee, a howl of indignation and wrath is heard throughout the country. Shame! We do not favor exagerated allowances; but if they are to be tolerated in any case, why not to a doctor as well as to anybody else?

THE Southern Dental Association will assemble the present year at Baltimore, August 8th next.

DEATH FROM CHLOROFORM.—Mr. George Allen, a citizen of Birmingham, Ala., died on June the 2d from inhaling chloroform to have a tooth extracted.

